# Identification of differentially expressed genes associated with the pathogenesis of gastric cancer by bioinformatics analysis

**DOI:** 10.1186/s12920-023-01720-7

**Published:** 2023-12-01

**Authors:** Fatemeh Abdolahi, Ali Shahraki, Roghayeh Sheervalilou, Sedigheh Sadat Mortazavi

**Affiliations:** 1 Department of Biology, Faculty of Science, University of Sistan and Baluchestan, Zahedan, Iran; 2 Pharmacology Research Center, Zahedan University of Medical Sciences, Zahedan, Iran; 3 Department of Biology, Faculty of Sciences, Shahid Bahonar University of Kerman, Kerman, Iran

**Keywords:** Differentially expressed genes (DEGs), Gastric cancer (GC), Bioinformatics, microRNA, Biomarkers

## Abstract

**Aim:**

Gastric cancer (GC) is one of the most diagnosed cancers worldwide. GC is a heterogeneous disease whose pathogenesis has not been entirely understood. Besides, the GC prognosis for patients remains poor. Hence, finding reliable biomarkers and therapeutic targets for GC patients is urgently needed.

**Methods:**

GSE54129 and GSE26942 datasets were downloaded from Gene Expression Omnibus (GEO) database to detect differentially expressed genes (DEGs). Then, gene set enrichment analyses and protein-protein interactions were investigated. Afterward, ten hub genes were identified from the constructed network of DEGs. Then, the expression of hub genes in GC was validated. Performing survival analysis, the prognostic value of each hub gene in GC samples was investigated. Finally, the databases were used to predict microRNAs that could regulate the hub genes. Eventually, top miRNAs with more interactions with the list of hub genes were introduced.

**Results:**

In total, 203 overlapping DEGs were identified between both datasets. The main enriched KEGG pathway was “Protein digestion and absorption.” The most significant identified GO terms included “primary alcohol metabolic process,” “basal part of cell,” and “extracellular matrix structural constituent conferring tensile strength.” Identified hub modules were COL1A1, COL1A2, TIMP1, SPP1, COL5A2, THBS2, COL4A1, MUC6, CXCL8, and BGN. The overexpression of seven hub genes was associated with overall survival. Moreover, among the list of selected miRNAs, hsa-miR-27a-3, hsa-miR-941, hsa-miR-129-2-3p, and hsa-miR-1-3p, were introduced as top miRNAs targeting more than five hub genes.

**Conclusions:**

The present study identified ten genes associated with GC, which may help discover novel prognostic and diagnostic biomarkers as well as therapeutic targets for GC. Our results may advance the understanding of GC occurrence and progression.

**Supplementary Information:**

The online version contains supplementary material available at 10.1186/s12920-023-01720-7.

## Introduction

Gastric cancer (GC) has been reported as the fifth most diagnosed cancer worldwide, with more than 1 million newly diagnosed cases annually. Also, GC is considered one of the leading causes of death by cancer all over the world [1]. Despite all improvements in treating GC, survival rates for GC patients remain unsatisfying and depend on the disease diagnosed stage [1, 2]. While the five-year survival rate is about less than 30% in most GC cases with advanced stages [3, 4], it decreases to less than 5% in patients diagnosed with the distant disease [1]. However, since most patients are diagnosed at an advanced stage, they lose the chance of the most effective surgical intervention, the gold standard for GC therapy [5, 6]. Hence, screening, early diagnosis, and targeted therapies are essential to boost the survival rate of its patients [5]. The main reason for late diagnosis in GC patients is the lack of sensitive and precise predictive markers for diagnosis [7, 8]. Since diagnosing GC cases relies on invasive approaches such as endoscopy and biopsy, finding sufficient non-invasive tests and biomarkers for GC screening or diagnosis is necessary [5].

GC is a heterogeneous disease with phenotypic diversity [9]. A variety of genetic and epigenetic alterations have been reported associated with gastric precancerous lesions and GC [10]. A growing body of literature focused on the potential of microRNAs (miRNAs or miRs) as novel biomarkers and therapeutic targets for GC patients [2, 11, 12]. MiRNAs are a subclass of small non-coding RNAs regulating gene expression post-transcriptionally [13, 14]. Aberrantly expressed miRNAs are associated with the onset and progression of various cancers, like GC [15]. Therefore, miRNAs are studied as attractive biomarker candidates for diagnosis and prognosis, as well as predictors of drug responses.

Nowadays, microarray and sequencing-based technologies have facilitated the discovery of genes and underlying mechanisms of tumors, as well as the exploration of biomarkers, prognostic factors, and therapeutic targets for a variety of cancers [16, 17]. Moreover, in recent years, a growing body of literature has focused on investigating new therapeutic targets and diagnostic markers for diseases such as cancers through employing bioinformatics analysis [18, 19]. However, although several studies have focused on identifying genes, miRNAs, and their interactions in GC through bioinformatics methods [20–23], it is still far from enough to reveal and understand the underlying pathogenesis of the disease.

Hence, the present study aims to find the essential genes and miRNAs participating in GC by employing bioinformatics methods and public database resources. In this regard, we first identified the differentially expressed genes (DEGs) in GC from microarray datasets derived from the GEO database. We comprehensively analyzed the identified DEGs. First, we performed Gene Ontology (GO) annotation and Kyoto Encyclopedia of Genes and Genomes (KEGG) pathway enrichment analyses. Then, after constructing a protein-protein interaction (PPI) network, we searched for hub modules among the DEGs. Afterward, we explored the expression distribution and prognostic significance of the selected hub genes. Eventually, after predicting miRNAs targeting the identified hub genes, we constructed the network of miRNA-hub genes.

We hope the results of our study provide a theoretical basis for the discovery of promising biomarkers and therapeutic targets to improve the clinical diagnosis and treatment of GC.

## Materials and methods

### Data collection from GEO repository

The GEO is a public repository containing high-throughput functional genomic data [24]. The GEO database (http://www.ncbi.nlm.nih.gov/geo) was investigated to find suitable gene expression datasets, employing the following customized criteria: ‘Gastric cancer’ and ‘Healthy control’ as keywords, ‘Homo sapiens’ as the organism, ‘Expression profiling by array’ as the study type, and studies with sample count ‘Higher than 20’. Eventually, two datasets, GSE54129 and GSE26942, were selected for further examinations. Microarray data of GSE54129 was obtained from the GPL570 platform ([HG-U133_Plus_2] Affymetrix Human Genome U133 Plus 2.0 Array) and collected from 111 gastric cancer tissues and 21 controls, comprised of biopsy normal gastric mucosa obtained from 21 volunteers underwent gastroscopy for health examinations. The GSE26942 data was based on the GPL6947 platform (Illumina HumanHT-12 V3.0 expression beadchip) and came from 206 GC patients and 12 normal samples.

### Screening DEGs in GC

R software version 4.01 was used for screening and finding DEGs in the selected datasets. R packages employed to accomplish this purpose were Limma 3.48.3, data.Table 1.14.2, plyr 1.8.6, BiocGenerics 0.40.0, BioBase 2.54, and ggplot 3.3.5. Moreover, the “EnhancedVolcano” package was utilized to draw volcano plots. Significant DEGs were defined as upregulated DEGs with logFC ≥ 1 and downregulated DEGs with logFC ≤ − 1, with an adjusted p-value < 0.001.Then, the overlapping DEGs in the selected datasets were identified via a Venn diagram created by an online Venn diagram maker available at https://bioinformatics.psb.ugent.be/webtools/Venn.

### Gene set enrichment analysis

To determine the biological implication of the overlapping DEGs, gene set enrichment analysis was performed using R software and employing several packages, including DOSE 3.20, org.Hs.eg.db 3.14.0, clusterProfiler 4.2.0. Results with a p.value < 0.05 were accepted as significant data.

### Protein-protein interaction analysis

STRING (http://string.embl.de/) is a database designed for constructing PPI networks and analyzing the functional interactions among proteins [25, 26]. The PPI network of the identified DEGs was obtained from the STRING biological database and then visualized through Cytoscape software (version 3.7.2 [27]). Furthermore, the plugin of the CytoHubba 0.1 of Cytoscape software was applied to the obtained results to explore the top hub genes in the constructed network of DEGs based on their score calculated via the degree method. Eventually, the PPI network of top hub modules was constructed.

### Validating the expression of the hub genes in GC

GEPIA 2.0 (available at http://gepia2.cancer-pku.cn/) is a cancer-specific database designed to analyze data based on TCGA and the Genotype-Tissue Expression (GTEx) databases [28]. Here, the GEPIA2 database was used to examine and validate the expression levels of the identified hub genes between GC and normal samples through the “Expression DIY” page of the database.

UALCAN (available at http://ualcan.path.uab.edu/analysis.html) is a web portal for conducting in-depth analyses of TCGA gene expression data [29, 30]. Here, the UALCAN database was employed to explore the differences in expression levels of each identified hub gene at various GC stages. A P-value < 0.05 were chosen as the p-value threshold for significant data.

### Survival analysis

The Kaplan–Meier plotter is a web-based tool available at (http://kmplot.com/analysis/). This database is suitable for investigating the prognostic values of genes in samples from various tumor types, including GC [31]. The Kaplan–Meier plotter was applied to examine the correlation between the effect of the key genes and overall survival in GC patients. The database can calculate the hazard ratio (HR) with a 95% confidence interval (95% CI) and log-rank p.value. In this step, a p.value < 0.05 were set as a threshold to distinguish statistically significant results.

### Identifying gene–miRNA interaction

Two online databases, including DIANA-TarBase v8 [32] and miRTarBase [33], were used to investigate the miRNAs regulating the hub genes. Both databases contain the experimentally validated associations between miRNAs and mRNAs [32, 33]. The lists of identified gene–miRNA interactions were merged for each hub gene to detect a single list of all valid interactions for the individual gene. Then, an online Venn diagram maker (https://bioinformatics.psb.ugent.be/webtools/Venn/) was used to find overlapping miRNAs. The miRNAs targeting more than four genes of the hub list were selected. Eventually, the final gene–miRNA interactions were visualized using Cytoscape software.

## Results

### Identification of DEGs in GC samples

**Fig. 1:**
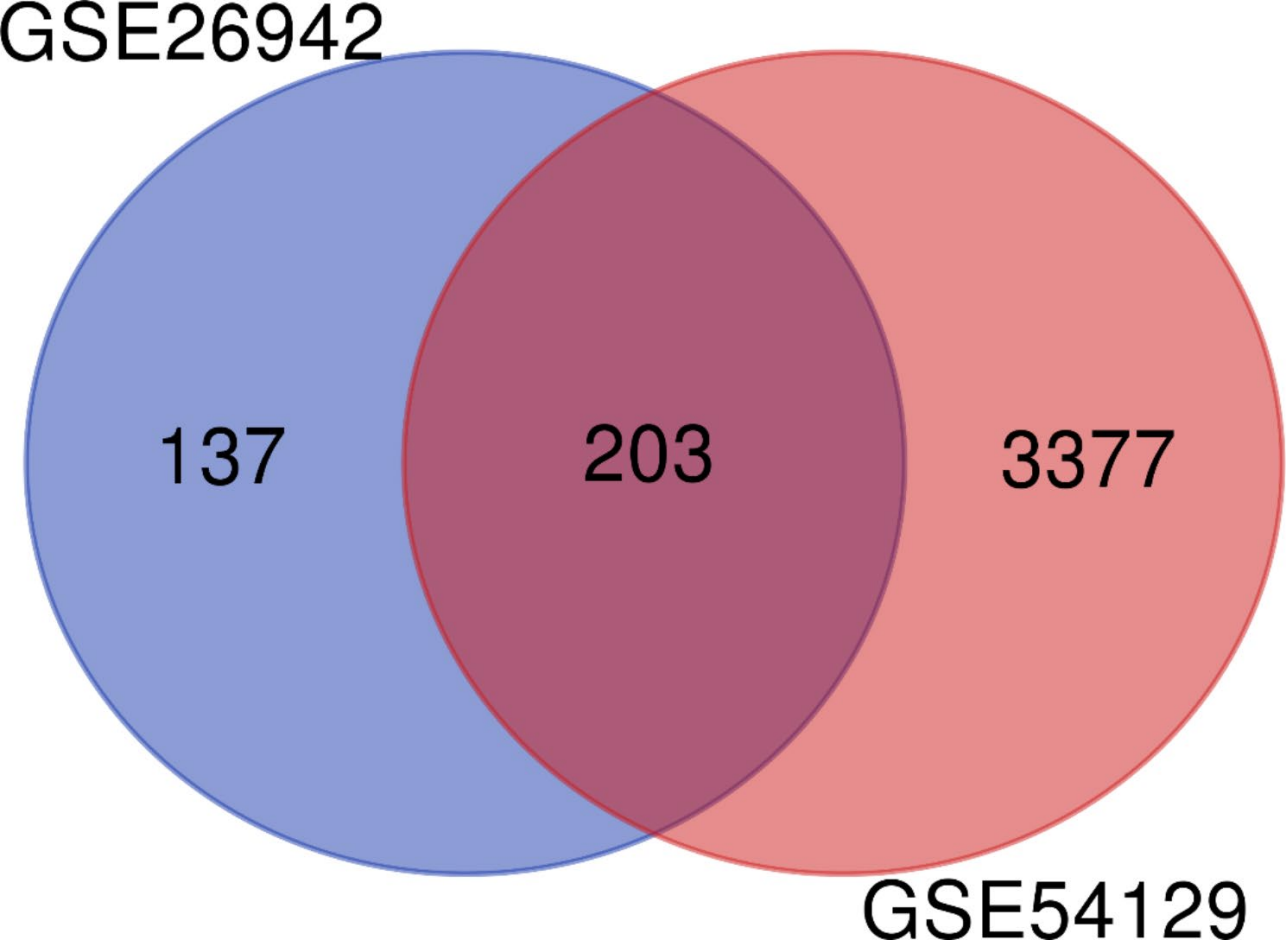
Venn diagram of the overlapping DEGs 203 common DEGs were detected between the two datasets (GSE54129 and GSE26942) using Venn diagram software (http://bioinformatics.psb.ugent.be/webtools/Venn/). DEGs = Differentially Expressed Genes

**Fig. 2:**
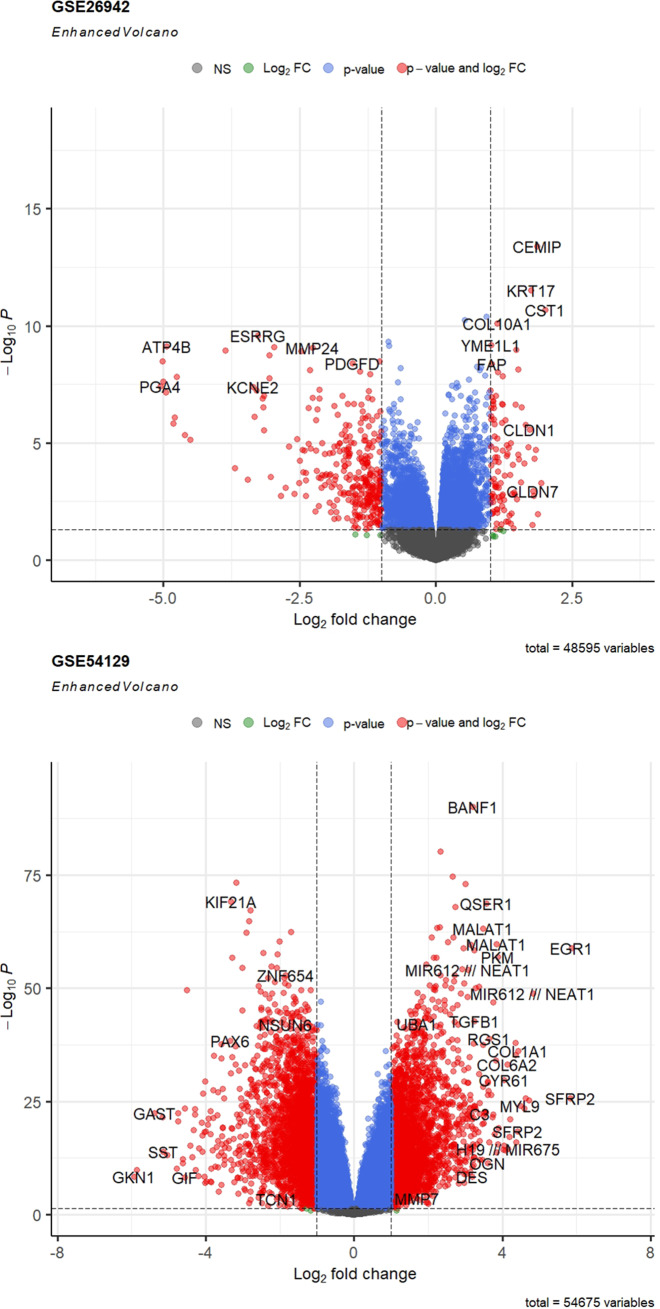
Volcano plots of DEGs in each GC dataset. Volcano Plot depicting DEGs between GC and control samples from GSE54129 and GSE26942 discriminated based on p-value and log2 fold-change. Colored dots represented genes with significant expression differences based on p-value (blue dots), only logFC (green dots), both p-value and log2 FC (red dots), or not significant in both terms (grey dots)

Two expression profiles (GSE54129 and GSE26942) were selected from the GEO database. A total of 317 GC tumors and 33 normal samples were obtained in this study. Employing the R software determined 3580 and 340 DEGs from GSE54129, and GSE26942, respectively. The Venn diagram identified 203 overlapping DEGs between selected datasets (Fig. 1). The lists of DEGs were presented in the supplementary file. Also, the volcano plots of the obtained DEGs from each dataset were drawn and illustrated in Fig. 2.

### GO and pathway analysis of overlapping DEGs

**Fig. 3:**
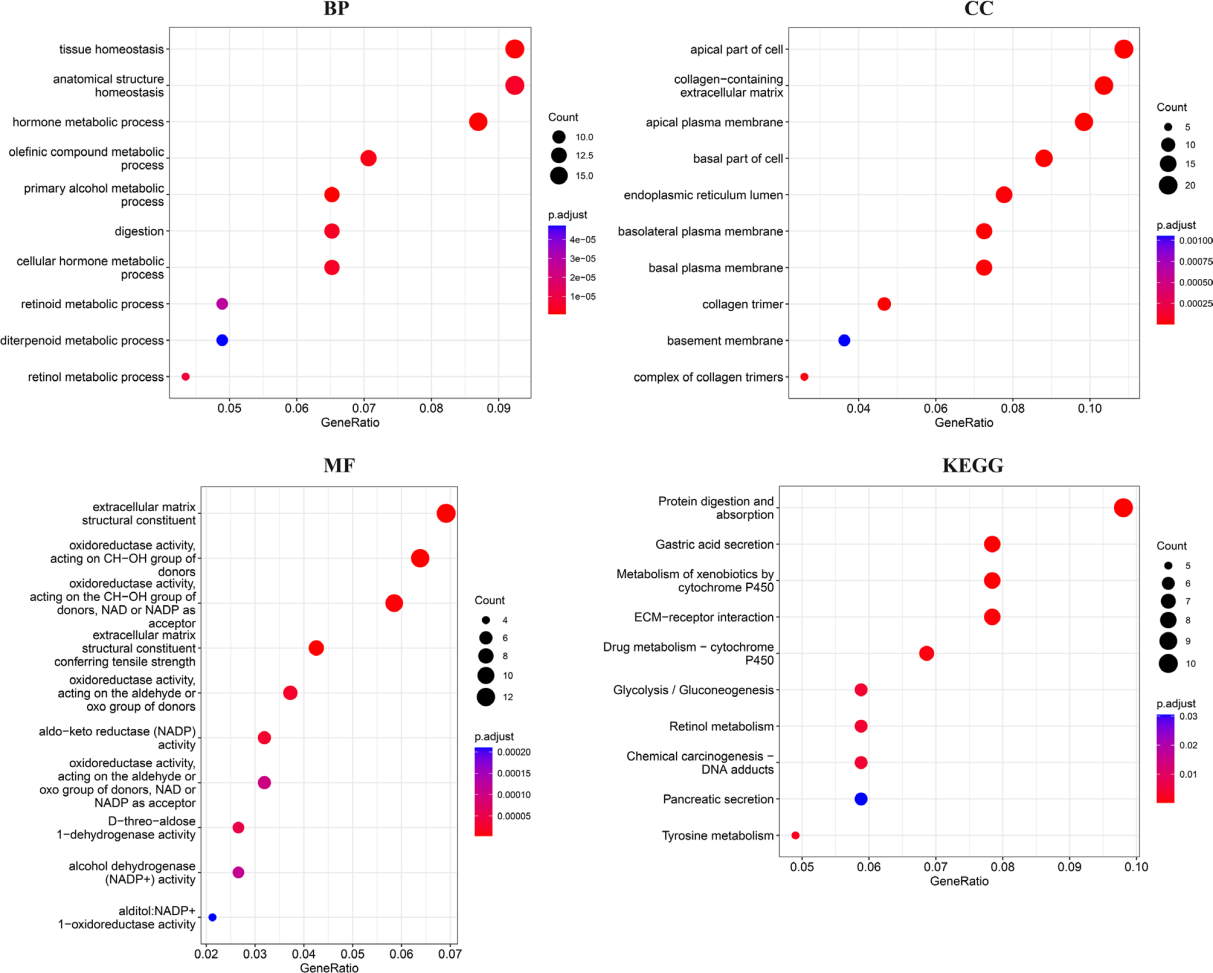
Gene Ontology and Pathway Analysis of overlapping DEGs. The most significant terms of BP, CC, MF, and KEGG pathways for overlapping DEGs were discovered and visualized using R software. The terms with a p.value and adjusted p.value < 0.05 were considered significant

In this step, GO, and KEGG pathway enrichment analyses of the overlapping DEGs were performed through R language. Based on the pathway analysis results, DEGs were particularly enriched in “Protein digestion and absorption” (P.value: 5.45E-07 and Gene count: 10), “Gastric acid secretion” (P.value: 4.28E-06 and Gene count: 8), “Metabolism of xenobiotics by cytochrome P450” (P.value: 5.21E-06 and Gene count: 8). Top identified pathways were presented in Table 1; Fig. 3. The results of GO enrichment analysis of 203 DEGs determined the most significant GO terms in biological process (BP), cellular component (CC), and molecular function (MF) as “primary alcohol metabolic process” (P.value: 3.54E-10 and Gene count: 12), “basal part of cell” (P.value: 2.27E-09 and Gene count: 17), and “extracellular matrix structural constituent conferring tensile strength” (P.value: 7.43E-09 and Gene count: 8), respectively. Other BP, CC, and MF were obtained in this study. The most significant terms of CC, BP, and MF were presented in Tables 2, 3 and 4; Fig. 3. Other results of GO and KEGG pathway enrichment analyses were presented in the supplementary file.

**Table 1:** The most significant KEGG pathways based on P-value*

ID	Description	Count	P-value	geneID
hsa04974	Protein digestion and absorption	10	5.45E-07	COL18A1, SLC7A8, CPA2, COL10A1, COL8A1, COL6A3, COL5A2, COL4A1, COL1A2, COL1A1
hsa04971	Gastric acid secretion	8	4.28E-06	KCNE2, SST, KCNJ16, CHRM3, CCKBR, CA2, ATP4B, ATP4A
hsa00980	Metabolism of xenobiotics by cytochrome P450	8	5.21E-06	SULT2A1, GSTA1, CYP3A5, ALDH3A1, ADH7, ADH1C, ADH1A, AKR7A3
hsa04512	ECM-receptor interaction	8	1.29E-05	THBS4, THBS2, SPP1, ITGA5, COL6A3, COL4A1, COL1A2, COL1A1
hsa00982	Drug metabolism - cytochrome P450	7	2.97E-05	MAOA, GSTA1, CYP3A5, ALDH3A1, ADH7, ADH1C, ADH1A
hsa00350	Tyrosine metabolism	5	7.73E-05	MAOA, ALDH3A1, ADH7, ADH1C, ADH1A
hsa00010	Glycolysis	6	0.003719	FBP2, ALDOB, ALDH3A1, ADH7, ADH1C, ADH1A
hsa00830	Retinol metabolism	6	0.000194	CYP3A5, CYP2C18, ALDH1A1, ADH7, ADH1C, ADH1A
hsa05204	Chemical carcinogenesis - DNA adducts	6	0.000211	SULT2A1, PTGS2, GSTA1, AKR1C2, CYP3A5, CYP2C18
hsa04972	Pancreatic secretion	6	0.001697	SLC12A2, RAB27B, PLA2G2A, CPA2, CHRM3, CA2
hsa04510	Focal adhesion	8	003609401	THBS4, THBS2, SPP1, ITGA5, COL6A3, COL4A1, COL1A2, COL1A1

*P-value < 0.05 was considered significant

**Table 2:** The most significant BP terms based on P-value*

BP terms	P-value	Count	geneID
primary alcohol metabolic process	3.54E-10	12	AKR1B10, AKR1C3, SULT2A1, SCNN1B, AKR1C2, CYP3A5, CYP2C18, AKR1C4, ALDH1A1, ADH7, ADH1C, ADH1A
hormone metabolic process	4.95E-10	16	AKR1B10, AKR1C3, SULT2A1, SPP1, SCNN1B, KLK6, FOXA1, AKR1C2, CYP3A5, CYP2C18, CTSL, AKR1C4, ALDH1A1, ADH7, ADH1C, ADH1A
tissue homeostasis	1.32E-09	17	VSIG1, CLDN18, SLC28A2, CLDN1, TFF2, TFF1, SPP1, SLC12A2, PTGS2, PIGR, MUC6, GCNT2, FOXC1, CLDN3, CDH3, CA2, ALDH1A1
olefinic compound metabolic process	3.77E-09	13	AKR1B10, AKR1C3, SCNN1B, PTGS2, GSTA1, AKR1C2, CYP3A5, CYP2C18, AKR1C4, ALDH1A1, ADH7, ADH1C, ADH1A
Digestion	1.08E-08	12	CAPN8, VSIG1, GKN1, CAPN9, TFF2, TFF1, SST, PGC, MUC6, AKR1C2, CHRM3, CCKBR
cellular hormone metabolic process	1.08E-08	12	AKR1B10, AKR1C3, SPP1, SCNN1B, AKR1C2, CYP3A5, CYP2C18, AKR1C4, ALDH1A1, ADH7, ADH1C, ADH1A
anatomical structure homeostasis	1.42E-08	17	VSIG1, CLDN18, SLC28A2, CLDN1, TFF2, TFF1, SPP1, SLC12A2, PTGS2, PIGR, MUC6, GCNT2, FOXC1, CLDN3, CDH3, CA2, ALDH1A1
retinol metabolic process	2.82E-08	8	AKR1B10, AKR1C3, CYP3A5, CYP2C18, ALDH1A1, ADH7, ADH1C, ADH1A
retinoid metabolic process	9.98E-08	9	AKR1B10, AKR1C3, CYP3A5, CYP2C18, AKR1C4, ALDH1A1, ADH7, ADH1C, ADH1A
diterpenoid metabolic process	1.87E-07	9	AKR1B10, AKR1C3, CYP3A5, CYP2C18, AKR1C4, ALDH1A1, ADH7, ADH1C, ADH1A

*P-value < 0.05 was considered significant

**Table 3:** The most significant CC terms based on P-value*

CC terms	P-value	Count	geneID
basal part of cell	2.27E-09	17	VSIG1, GKN2, PROM2, SLC7A8, HEPH, CLDN1, SLC12A2, REG1A, LEPR, KCNJ16, HPGD, FAP, CHRM3, CEACAM5, CA9, CA2, AQP4
apical part of cell	2.28E-09	21	PROM2, MUC17, SLC26A9, SLC44A4, SLC7A8, CLDN1, SORBS2, THY1, SLC12A2, SCNN1G, SCNN1B, SCNN1A, RAB27B, MUC1, MAL, FAP, CTSL, CEACAM5, CA2, ATP4B, ATP4A
apical plasma membrane	4.44E-09	19	PROM2, MUC17, SLC26A9, SLC44A4, SLC7A8, CLDN1, SORBS2, THY1, SLC12A2, SCNN1G, SCNN1B, SCNN1A, RAB27B, MUC1, MAL, CTSL, CEACAM5, ATP4B, ATP4A
collagen-containing extracellular matrix	8.63E-09	20	MUC17, CTHRC1, COL18A1, NTN4, SULF1, TIMP1, THBS4, THBS2, SERPINE2, SERPINE1, LGALS1, CTSL, COL10A1, COL8A1, COL6A3, COL5A2, COL4A1, COL1A2, COL1A1, BGN
basolateral plasma membrane	7.59E-08	14	VSIG1, PROM2, SLC7A8, HEPH, CLDN1, SLC12A2, LEPR, KCNJ16, HPGD, CHRM3, CEACAM5, CA9, CA2, AQP4
collagen trimer	1.95E-07	9	CTHRC1, COL18A1, COL10A1, COL8A1, COL6A3, COL5A2, COL4A1, COL1A2, COL1A1
basal plasma membrane	2.81E-07	14	VSIG1, PROM2, SLC7A8, HEPH, CLDN1, SLC12A2, LEPR, KCNJ16, HPGD, CHRM3, CEACAM5, CA9, CA2, AQP41
endoplasmic reticulum lumen	5.41E-07	15	COL18A1, PDIA2, MZB1, TIMP1, SPP1, PTGS2, LGALS1, COL10A1, COL8A1, COL6A3, COL5A2, COL4A1, COL1A2, COL1A1, ARSD
complex of collagen trimers	1.59E-06	5	COL8A1, COL5A2, COL4A1, COL1A2, COL1A1
basement membrane	4.66E-05	7	COL18A1, NTN4, TIMP1, THBS4, THBS2, COL8A1, COL4A1

*P-value < 0.05 was considered significant

**Table 4:** The most significant MF terms based on P-value*

MF terms	P-value	Count	geneID
extracellular matrix structural constituent conferring tensile strength	7.43E-09	8	COL18A1, COL10A1, COL8A1, COL6A3, COL5A2, COL4A1, COL1A2, COL1A1
oxidoreductase activity, acting on CH-OH group of donors	1.56E-08	12	AKR1B10, AKR7A3, PTGR1, AKR1C3, LIPF, HPGD, AKR1C2, AKR1C4, ALDH3A1, ADH7, ADH1C, ADH1A
extracellular matrix structural constituent	2.52E-08	13	MUC17, CTHRC1, COL18A1, THBS2, MUC6, COL10A1, COL8A1, COL6A3, COL5A2, COL4A1, COL1A2, COL1A1, BGN
oxidoreductase activity, acting on the CH-OH group of donors, NAD or NADP as acceptor	5.97E-08	11	AKR1B10, AKR7A3, PTGR1, AKR1C3, HPGD, AKR1C2, AKR1C4, ALDH3A1, ADH7, ADH1C, ADH1A
oxidoreductase activity, acting on the aldehyde or oxo group of donors	2.93E-07	7	AKR1B10, AKR1C3, ALDH6A1, AKR1C4, ALDH3A1, ALDH1A1, ADH7
aldo-keto reductase (NADP) activity	4.15E-07	6	AKR1B10, AKR7A3, AKR1C3, AKR1C2, AKR1C4, ALDH3A1
D-threo-aldose 1-dehydrogenase activity	8.19E-07	5	AKR1B10, AKR7A3, AKR1C3, AKR1C2, AKR1C4
oxidoreductase activity, acting on the aldehyde or oxo group of donors, NAD or NADP as acceptor	1.90E-06	6	AKR1B10, AKR1C3, ALDH6A1, AKR1C4, ALDH3A1, ALDH1A1
alcohol dehydrogenase (NADP+) activity	2.44E-06	5	AKR1B10, AKR1C, AKR1C2, AKR1C4, ALDH3A1
alditol:NADP + 1-oxidoreductase activity	4.94E-06	4	AKR1B10, AKR1C3, AKR1C2, AKR1C4

*P-value < 0.05 was considered significant

### PPI networks

**Fig. 4:**
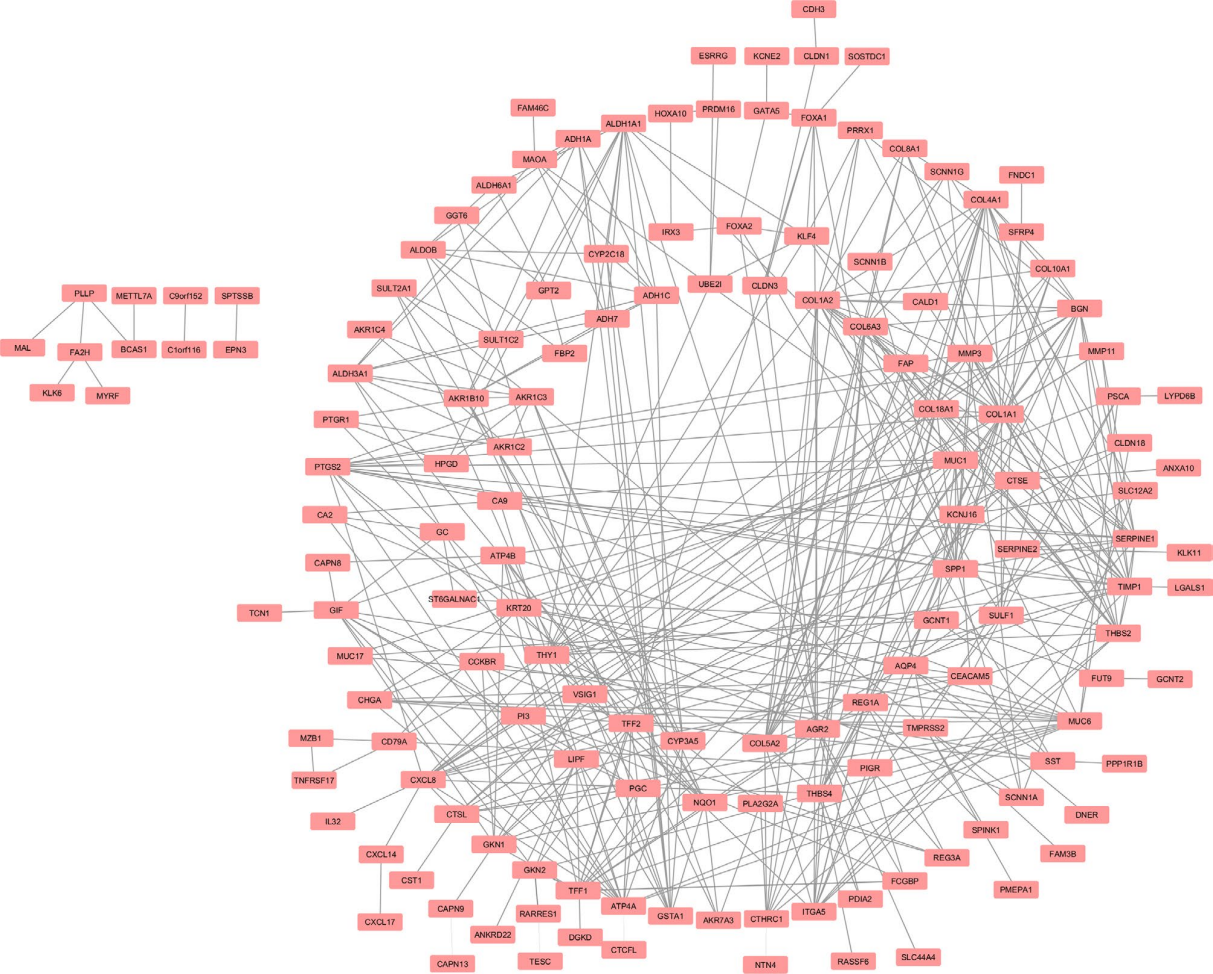
PPI Network of overlapping DEGs. The PPI network with 150 nodes and 416 edges was constructed via Cytoscape

**Fig. 5:**
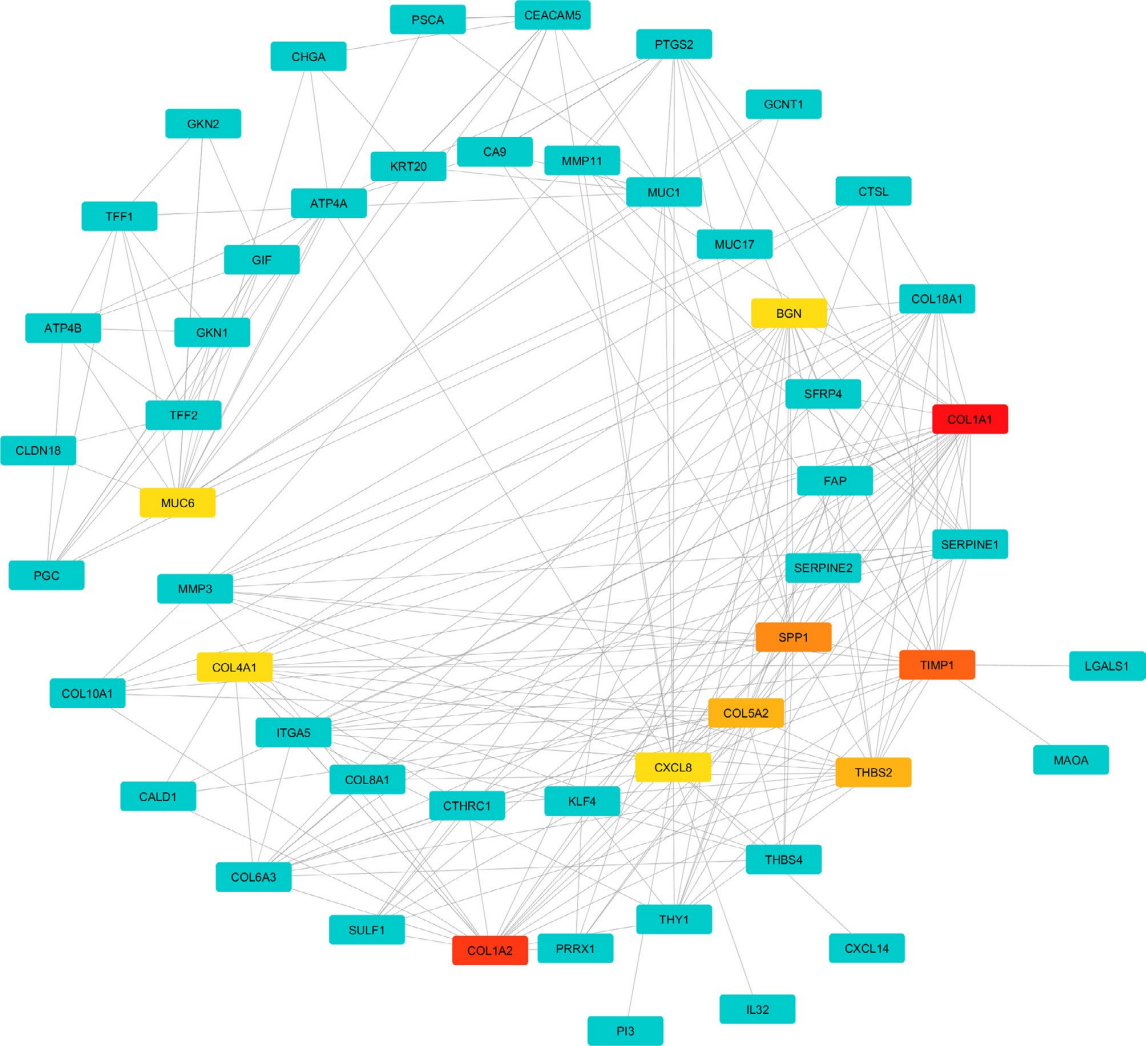
PPI Network of top 10 hub genes. The PPI network with 52 nodes and 220 edges was constructed via Cytoscape

At this step, we used 203 DEGs to construct a PPI network utilizing the STRING database and Cytoscape. The obtained network with 150 nodes and 416 edges is presented in Fig. 4. Subsequently, using the CytoHubba plugin, the ten hub genes, including COL1A1, COL1A2, TIMP1, SPP1, COL5A2, THBS2, COL4A1, MUC6, CXCL8, and BGN, were identified. A network of hub genes was built with 52 nodes and 220 edges (Fig. 5; Table 5).

**Table 5:** Top 10 hub genes in network Ranked by Degree method

Rank	Name	Ensembl	Score
1	COL1A1	ENSG00000108821	25
2	COL1A2	ENSG00000164692	20
3	TIMP1	ENSG00000102265	19
4	SPP1	ENSG00000118785	18
5	COL5A2	ENSG00000204262	17
5	THBS2	ENSG00000186340	17
7	COL4A1	ENSG00000187498	16
7	MUC6	ENSG00000184956	16
7	CXCL8	ENSG00000169429	16
7	BGN	ENSG00000182492	16

### Validation of the gene expression

**Fig. 6:**
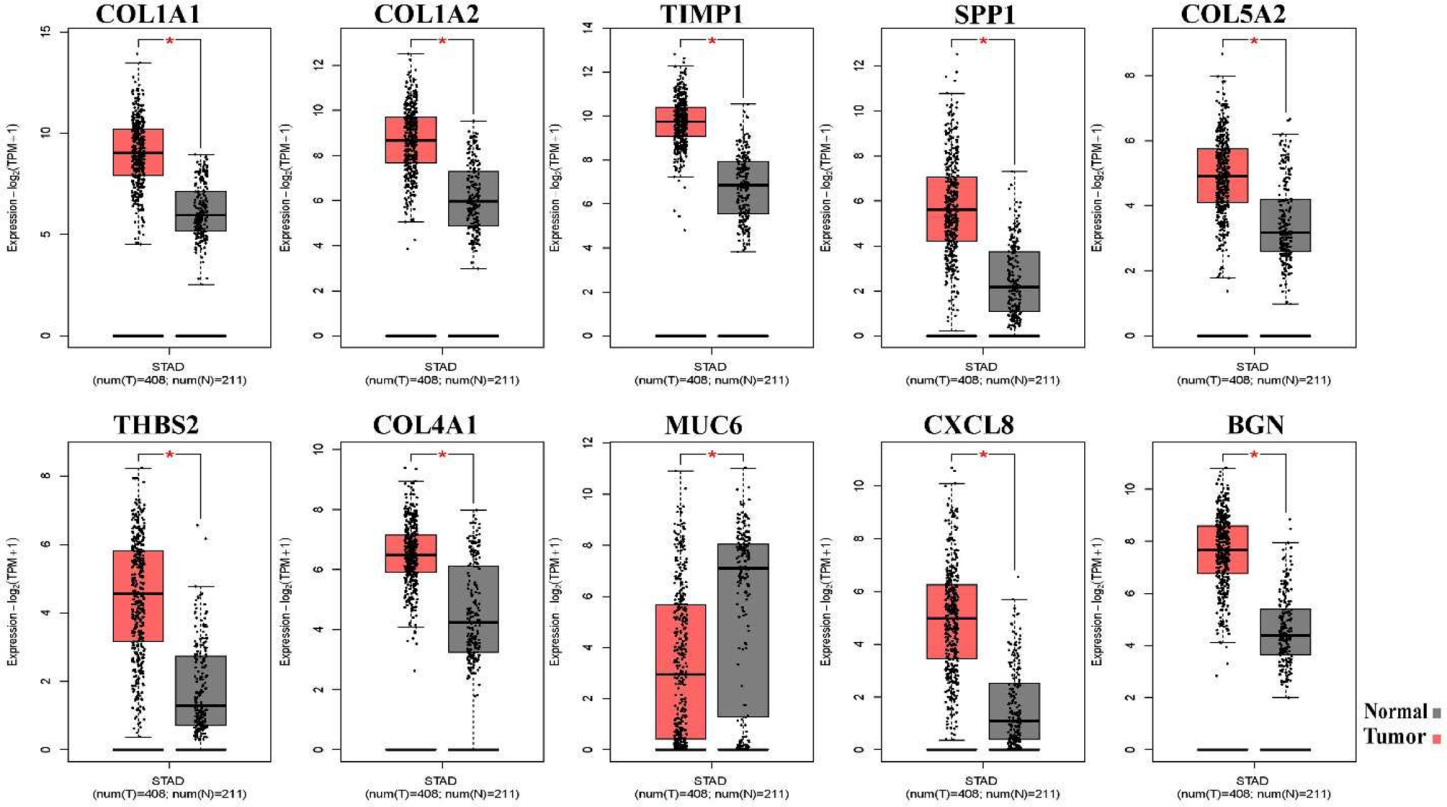
mRNA expression of identified hub genes. Comparison of expression levels of 10 identified hub genes, including COL1A1, COL1A2, TIMP1, SPP1, COL5A2, THBS2, COL4A1, MUC6, CXCL8, and BGN, in gastric cancer tissue (red; n = 408) and normal tissues (black; n = 211) using the GEPIA2 database. * P value < 0.05

In this step, GEPIA was employed to investigate the expression levels of selected key genes in GC patients and healthy samples. The GEPIA results confirmed our data and reflected that all the selected hub genes except MUC6 were over-expressed in GC samples compared to normal samples. MUC6 was expressed at lower levels in GC compared with normal gastric tissues. All obtained results were significant (P < 0.05; Fig. 6).

**Fig. 7:**
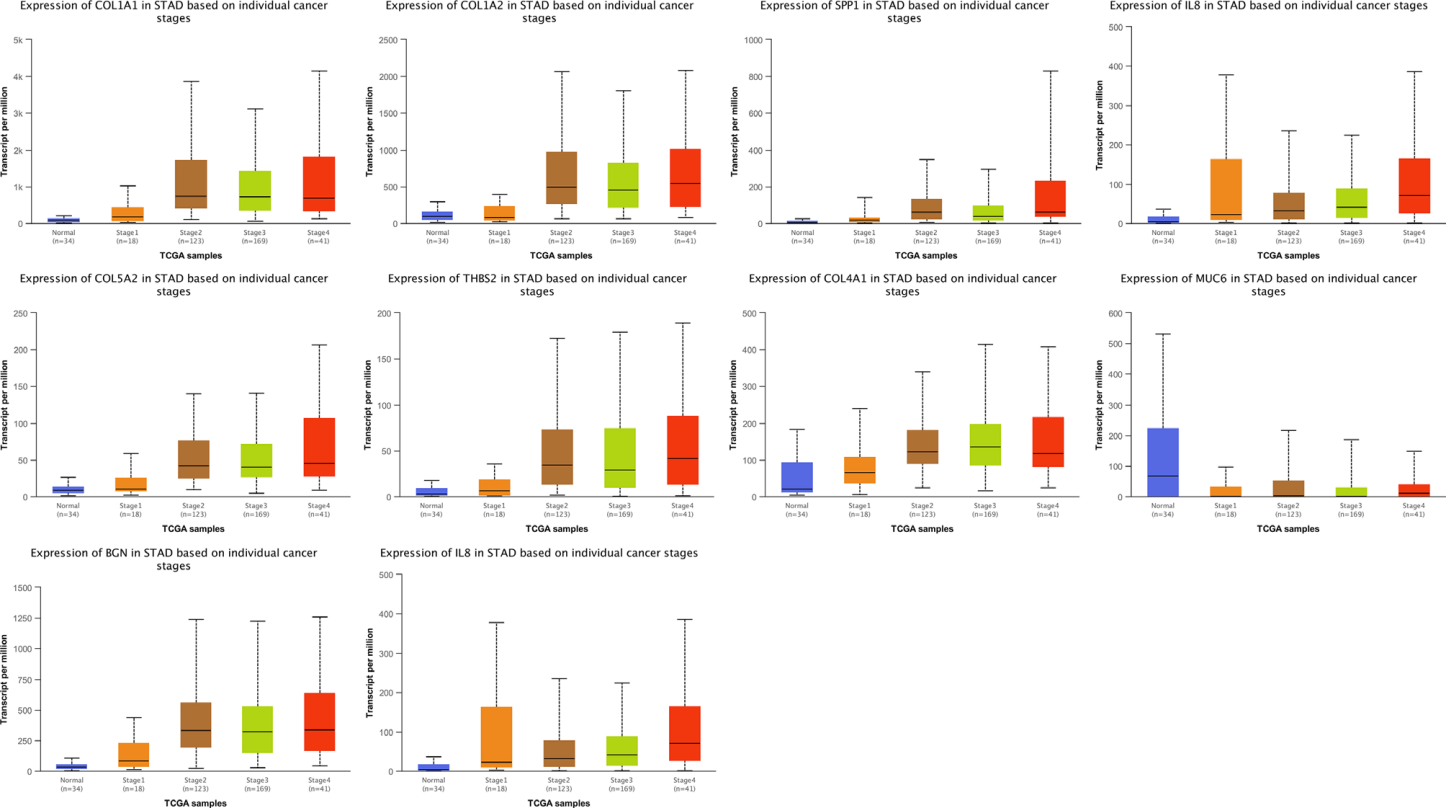
Correlation between the expression levels of each identified hub gene and various stages of gastric cancer. Box-whisker plots made by UALCAN showed the expression of hub genes, including COL1A1, COL1A2, TIMP1, SPP1, COL5A2, THBS2, COL4A1, CXCL8, and BGN, elevated in different stages of gastric cancer (stages 1, 2, 3, and 4) in TCGA samples. MUC6 is downregulated in the GC stages

Furthermore, the UALCAN results discerned that the expression pattern of the hub genes, including COL1A1, COL1A2, TIMP1, SPP1, COL5A2, THBS2, COL4A1, CXCL8, and BGN, were significantly higher in GC stages I–IV than normal samples, whereas MUC6 was significantly downregulated in different stages of GC (Fig. 7). The obtained results were consistent with the finding of the selected microarray datasets, indicating that the expression of all hub genes except MUC6 was increased in GC.

### Survival analysis of the hub genes

**Fig. 8:**
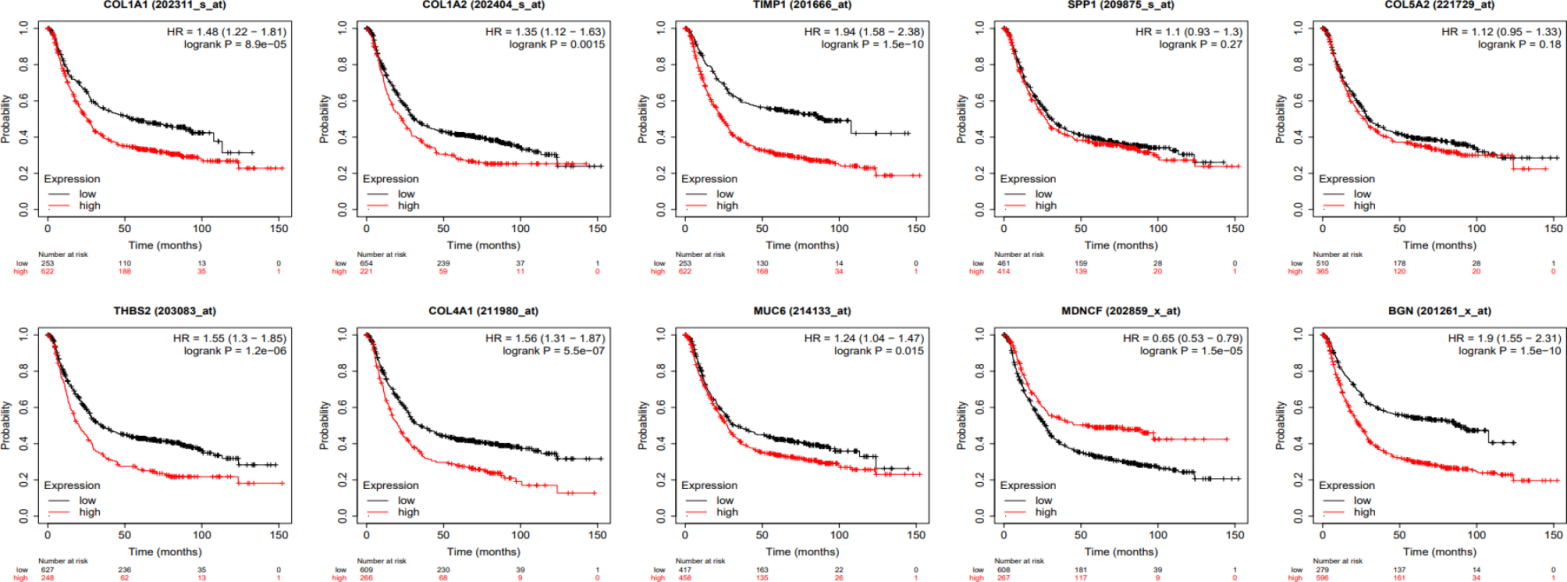
Kaplan–Meier overall survival analysis for the top 10 hub genes. High expression of *COL1A1, COL1A2, TIMP1, THBS2, COL4A1, MUC6, and BGN* was associated with poor overall survival of GC patients. On the other hand, high expression of *CXCL8* (aka *MDNCF*) was found to be associated with favorable overall survival. The expression of *COL5A2 and SPP1 was* not related to the overall survival of GC patients. GC: gastric cancer

Overall survival curves were drawn to investigate the prognostic values of the identified hub genes in 875 GC patients by using the Kaplan-Meier plotter. Based on the median expression of the candidate genes, patients were divided into two groups [1] those with a high expression level of the desired gene and [2] others with a low expression level of it. The analysis determined that eight of ten key genes were significantly associated with the prognosis of GC patients. Among them, high expressions of COL1A1 (P.value: 8.9E-5), COL1A2 (P.value: 0.0015), TIMP1 (P.value: 1.5E-10), THBS2 (P.value: 1.2E-6), COL4A1 (P.value: 5.5E-7), MUC6 (P.value: 0.0151), and BGN (P.value: 1.5E-10) were significantly correlated with poor overall survival probability for GC patients (Fig. 8). High expression of CXCL8 (aka MDNCF) was detected to be associated with favorable overall survival (P.value: 1.5E-5). However, COL5A2 (P.value: 0.1769) and SPP1 (P.value: 0.2713) had nonsignificant log-rank p values and were independent of the prognosis of GC patients (Fig. 8).

### Gene–miRNA interaction network

**Fig. 9:**
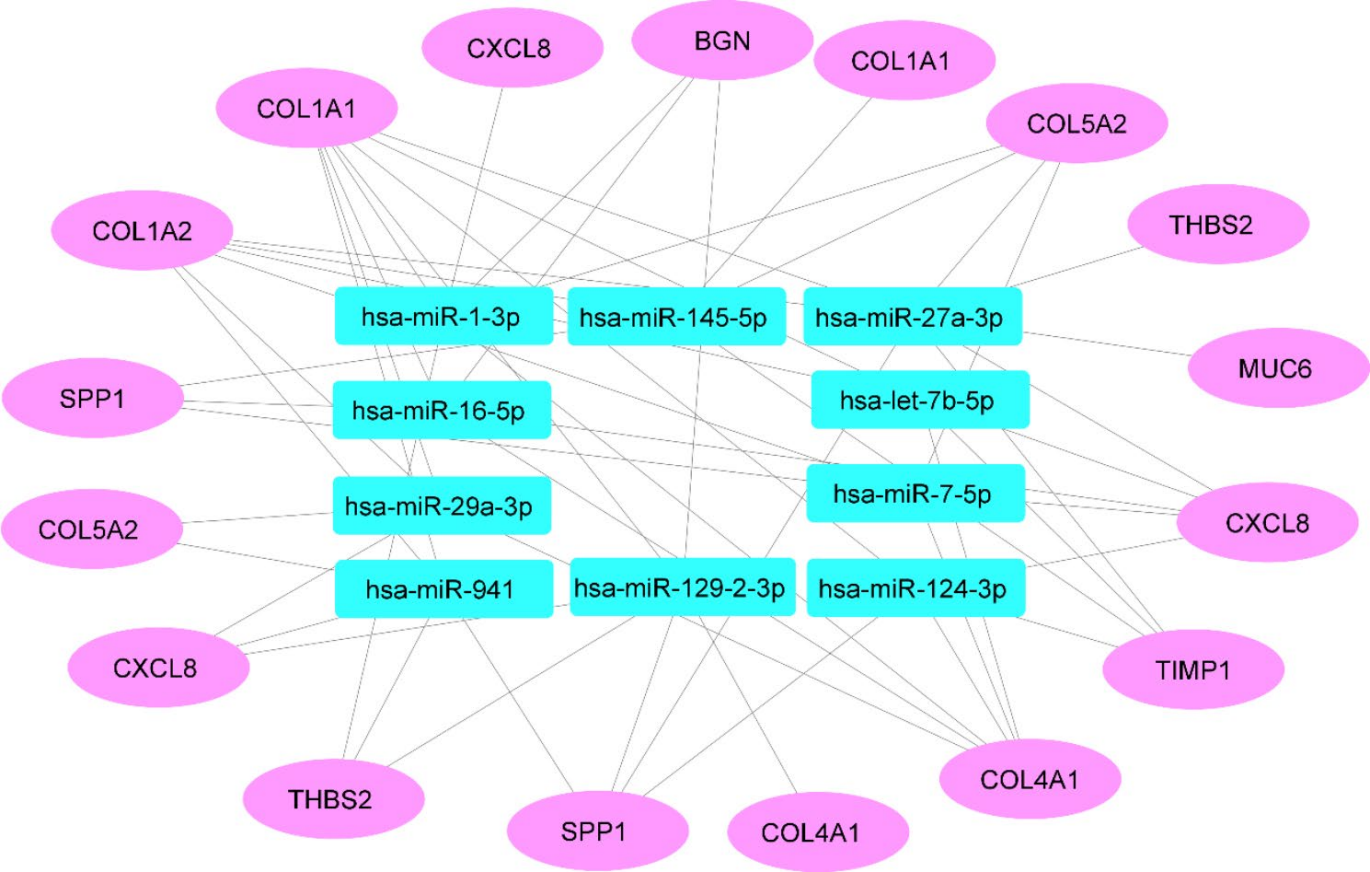
miRNA-gene interaction network. The miRNA-gene interaction network with 27 nodes and 56 edges was constructed via Cytoscape

DIANA-TarBase v8 and miRTarBase were investigated for the miRNAs regulating the hub genes. A total of 101, 109, 32, 30, 58, 134, 117, 15, 75, and 33 miRNA-gene interactions were found for COL1A1, COL1A2, TIMP1, SPP1, COL5A2, THBS2, COL4A1, MUC6, CXCL8, and BGN, respectively. After merging the lists of identified gene–miRNA interactions, a single list containing ten miRNAs targeting more than four hub genes was chosen, including hsa-miR-27a-3, hsa-miR-941, hsa-miR-129-2-3p, hsa-miR-1-3p, hsa-miR-145-5p, hsa-let-7b-5p, hsa-miR-29a-3p, hsa-miR-124-3p, hsa-miR-16-5p, and hsa-miR-7-5p (Table 6) (supplementary file). Four miRNAs, including hsa-miR-27a-3, hsa-miR-941, hsa-miR-129-2-3p, and hsa-miR-1-3p, were selected as top miRNAs since they interact with more than half of the identified hub genes. Moreover, Cytoscape was employed to visualize the miRNA-gene interactions in a network with 27 nodes and 56 edges (Fig. 9).

**Table 6:** Top Gene–miRNA Interactions

Gene	microRNA
COL1A1, COL1A2, COL5A2, CXCL8, MUC6, SPP1, THBS2, TIMP1	hsa-miR-27a-3p
COL1A1, COL1A2, COL5A2, CXCL8, SPP1, THBS2	hsa-miR-941
BGN, COL1A1, COL4A1, CXCL8, SPP1, THBS2	hsa-miR-129-2-3p
BGN, COL1A1, COL4A1, COL5A2, CXCL8, THBS2	hsa-miR-1-3p
COL1A1, COL1A2, COL5A2, SPP1, TIMP1	hsa-miR-145-5p
COL1A1, COL1A2, COL4A1, CXCL8, TIMP1	hsa-let-7b-5p
COL1A1, COL1A2, COL4A1, COL5A2, CXCL8	hsa-miR-29a-3p
COL1A1, COL4A1, CXCL8, SPP1, TIMP1	hsa-miR-124-3p
BGN, COL1A1, COL4A1, CXCL8, SPP1	hsa-miR-16-5p
COL1A2, COL4A1, COL5A2, CXCL8, SPP1	hsa-miR-7-5p

## Discussion

In spite of a gradual decrease in the incidence and mortality rate, GC is still considered a leading cause of cancer mortality worldwide [1]. Besides, the early diagnosis and survival rate are still unfavorable for this cancer [34]. Therefore, it is critical to understand the underlying mechanisms and to determine biomarkers for developing strategies for screening, early diagnosis, and novel therapies for GC [35]. Hence, finding novel prognostic factors and/or biomarkers for early detection is required to improve patient outcomes.

In this study, we identified 203 DEGs in two GEO datasets of GC, GSE54129 and GSE26942. GO, and KEGG pathway enrichment analyses determined “Protein digestion and absorption,” “primary alcohol metabolic process,” “basal part of cell,” and “extracellular matrix structural constituent conferring tensile strength” as the most significant cancer-related pathways, BP, CC, and MF terms in which DEGs were enriched. The STRING and Cytoscape were employed to construct the PPI network. The plugin of CytoHubba introduced the top 10 hub genes, including COL1A1, COL1A2, COL5A2, COL4A1, TIMP1, SPP1, THBS2, MUC6, CXCL8, and BGN. All identified hub genes, except COL5A2 and SPP1, were significantly correlated with the overall survival of GC patients. After verifying the expression of all hub genes in GC, the miRNA-mRNA interactions were predicted for them. Among identified miRNAs, hsa-miR-27a-3, hsa-miR-941, hsa-miR-129-2-3p, and hsa-miR-1-3p, selected as top miRNAs interacting with more than half of the hub gene list.

Our results revealed the implication of different collagen family members, including COL1A1, COL4A1, COL5A2, and COL4A1, in GC samples. As critical parts of the ECM component, the members of this family are closely related to tumor prognosis, proliferation, invasion, and drug resistance [36]. COL1A1 and COL1A2 are overexpressed in GC and promote cell proliferation, invasion, and migration [37–39]. Li et al. shed light on the potential of COL1A1 as a monitoring factor for screening early GC. Besides, their results revealed a link between the overexpression of COL1A1 and COL1A2 with a poor overall survival rate of GC [40]. Previously, other studies proposed COL1A1, COL1A2, and COL4A1 as candidate diagnostic markers for this cancer [41, 42]. COL4A1 has been suggested as a potential biomarker and inflammation-related target for GC [43]. In silico studies have shed light on the potential of COL4A1 in conferring trastuzumab resistance and promoting gastric carcinoma recurrence [44, 45]. Additionally, COL4A1 has been implicated in trastuzumab resistance in gastric cancer, potentially conferring resistance to this targeted therapy [46]. Biglycan (BGN) is another critical component of ECM proteins involved in the development and aggressiveness of GC [47, 48]. This gene may implicate GC progression and development through the chronic activating of tumor angiogenesis [48]. A link was detected between BGN overexpression and worse clinical and prognostic parameters of GC [47, 49]. The mechanism of BGN-induced gastric cancer involves the induction of epithelial to mesenchymal transition (EMT) and upregulation of chromatin reprogramming factors [50]. The tissue inhibitor of metalloproteinases 1 (TIMP1) is an important player in ECM remodeling [51]. Preoperative TIMP1 expression level in peripheral blood may link to the GC stage, suggesting its potential application as a marker for tumor invasion and metastasis [52]. Hence, the expression level of TIMP1 has been suggested as a clinical biomarker for the screening, diagnosis, and prognostic of GC [52–54]. An in-silico study identified a correlation between overexpression of COL4A1, TIMP1, and COL1A2 with worse overall survival in GC [55]. Secreted phosphoprotein 1 (SPP1), an acidic glycoprotein known as osteopontin (OPN), participated in EMT and tumor metastasis [56]. Research showed that SPP1 suppresses the Wnt/β-catenin pathway, thereby enhancing the radiosensitivity of GAC through inhibition of invasion and acceleration of DNA damage, G2/M phase arrest, and apoptosis [57]. Lu et al. using bioinformatics analysis identified that SPP1 and FN1 were upregulated in GC than normal samples in their selected datasets [58]. Another study demonstrated that type I collagen promoted TIC-like phenotypes and chemoresistance through ITGB1/YBX1/SPP1/NF-κB pathway [59]. Thrombospondin 2 (THBS2) a member of the thrombospondin family, has been suggested as an early diagnostic marker for patients with GC [60]. Wang et al. showed that while SPP1 had no significant association with GC overall survival, high expression level of THBS2 in GC patients was correlated with shorter survival time [61] [60].Bioinformatics reanalysis suggested the prognostic value of COL1A1, COL1A2, and THBS2 in GC. KEGG reanalysis determined that these genes, together with COL2A1 and COL11A1, were enriched in the ECM-receptor interaction pathways [62].MUC6, Musin 6, is a marker of gastric foveolar and antral mucous glandular cells that shows gastric phenotypes. literatures indicated that the expression of MUC6 was regulated by promoter methylation which lead to the downregulation of MUC6 in GC and induce the progression of GC [63]. Zheng et al. reported a link between the downregulation of MUC6 with progression, poor prognosis, and metastasis of GC [64]. Considering transcriptomics and single-cell sequencing, another study suggested BGN and COL5A2 as GC diagnostic and prognostic biomarkers applicable for predicting drug sensitivity in GC [49]. Despite controversial evidence regarding the link between the expression level of COL5A2 and the survival of GC patients [49, 65–68], Zhang et al. and Cao et al. confirmed our results by reporting no significant correlation between COL5A2 and the overall survival of GC patients [66, 69]. Studies have also demonstrated that serum CXCL8 levels can significantly predict GC risk and reveal a role of the CXCL8/CXCR2 axis and inflammation in the pathogenesis of this malignancy [70]. CXCL8, which is primarily secreted by macrophages in gastric cancer, is linked to poor clinical outcomes and tumor progression. CXCL8 promotes an immunosuppressive environment by increasing PD-L1 expression on macrophages, hindering CD8 + T-cell function, and limiting infiltration [71]. Several studies identified positive impacts of higher levels of CXCL8 expression on the overall survival of GC patients [72–75].

Several studies highlighted the role of hsa-miR-27a-3p as oncomiR upregulated in GC [76–78]. Moreover, miR-27a-3p/BTG2 axis was proposed as not only a promising diagnostic biomarker but also a potential therapeutic target for GC patients [77]. Previously, the link between overexpression of miR-27a and significant up-regulation of COL1A2 was reported in hepatic stellate cells [79]. An inverse correlation between miR-27a-3p and CXCL8 was reported [80]. MicroRNA-27a-3p negatively regulates SPP1 to inhibit lung and skin fibrosis of systemic sclerosis [81]. MiR-129-2-3p regulates cell proliferation in GC cells [82]. Gastric juice miR-129-2-3p has been suggested as a potential biomarker for the screening GC [83]. MiR-129-5p regulates GC invasion through interacting with IL-8 and COL1A1 [84, 85]. The interaction between COL1A1 and MiR-129-5p has been proposed as a potential therapeutic target for GC [86]. MiR-1-3p is another miRNA suppressing proliferation and invasion of GC cells [87]. Although the role of miR-941 has been investigated in several cancers, such as breast and prostate cancer [88, 89], its association has not been reported with GC. Previous studies supported our results and revealed the link between selected miRNAs and GC. However, at the time of this study, most of the miRNA-gene interactions identified in this study have not been investigated in GC.

The results of this study provide a more comprehensive understanding of the underlying mechanisms of GC and suggest novel biomarkers for prognosis and diagnosis, as well as therapeutic targets for GC patients. The most important limitation of our study is the lack of experimental work to validate the results obtained from bioinformatics methods. Therefore, further studies with larger sample sizes, animal models, and clinical tissue verification are required to confirm our results.

## Conclusion

In conclusion, using two datasets obtained from the GEO database and integrated bioinformatics analysis, ten GC-associated hub genes were found. Except for MUC6, the expression of other hub genes was revealed to be upregulated in GC. The overexpression of seven hub genes was associated with GC’s poor overall survival. Then, the miRNA-mRNA interactions were predicted for each hub gene. Although more experimental investigations with larger sample sizes are required to validate the findings of the present study, we hope that our results will assist in the discovery of novel biomarkers and therapeutic targets for GC and advance the understanding of its pathogenesis.

### Electronic supplementary material

Below is the link to the electronic supplementary material.

Supplementary Material 1

## Data Availability

All databases (including NCBI GEO, Veen Diagram maker, etc.) are Freely available on the web. The raw data of this study are obtained from the GEO database (available at https://www.ncbi.nlm.nih.gov/geo/).
